# Prevalence of intellectual and developmental disabilities among first generation adult newcomers, and the health and health service use of this group: A retrospective cohort study

**DOI:** 10.1371/journal.pone.0215804

**Published:** 2019-06-20

**Authors:** Anna Durbin, James K. H. Jung, Hannah Chung, Elizabeth Lin, Robert Balogh, Yona Lunsky

**Affiliations:** 1 Li Ka Shing Knowledge Institute, St. Michael’s Hospital, Toronto, Ontario, Canada; 2 Department of Psychiatry, University of Toronto, 1 King’s College Cir., Toronto, Ontario, Canada; 3 ICES, Toronto, Ontario, Canada; 4 Dalla Lana School of Public Health, University of Toronto, Toronto, Ontario, Canada; 5 Adult Neurodevelopmental Services, Centre for Addiction and Mental Health, W., Toronto, Ontario, Canada; 6 Provincial System Support Program, Centre for Addiction and Mental Health, Toronto, Ontario, Canada; 7 Faculty of Health Sciences, University of Ontario Institute of Technology, Oshawa, Ontario, Canada; New York City Department of Health and Mental Hygiene, UNITED STATES

## Abstract

**Background:**

Attention to research and planning are increasingly being devoted to newcomer health, but the needs of newcomers with disabilities remain largely unknown. This information is difficult to determine since population-level data are rarely available on newcomers or on people with intellectual and developmental disabilities (IDD), although in Ontario, Canada these databases are accessible. This study compared the prevalence of IDD among first generation adult newcomers to adult non-newcomers in Ontario, and assessed how having IDD affected the health profile and health service use of newcomers.

**Methods:**

This population-based retrospective cohort study of adults aged 19–65 in 2010 used linked health and social services administrative data. Prevalence of IDD among newcomers (n = 1,649,633) and non-newcomers (n = 6,880,196) was compared. Among newcomers, those with IDD (n = 2,830) and without IDD (n = 1,646,803) were compared in terms of health conditions, and community and hospital service use.

**Results:**

Prevalence of IDD was lower in newcomers than non-newcomers (171.6 versus 898.3 per 100,000 adults, p<0.0001). Among newcomers, those with IDD were more likely than those without IDD to have comorbid physical health disorders, non-psychotic, psychotic and substance use disorders. Newcomers with IDD were also more likely to have psychiatry visits, and frequent emergency department visits and hospitalizations.

**Conclusion:**

First generation adult newcomers have lower rates of IDD than non-newcomers. How much of this difference is attributable to admission policies that exclude people expected to be high health service users versus how much is attributable to our methodological approach is unknown. Finding more medical and psychiatric comorbidity, and more health service use among newcomers with IDD compared to newcomers without IDD is consistent with patterns observed in adults with IDD more generally. To inform polices that support newcomers with IDD future research should investigate reasons for the prevalence finding, barriers and facilitators to timely health care access, and pathways to care.

## Introduction

Across settings newcomers tend to have better health than non-newcomers (“Healthy Immigrant Effect”)[1]. They also tend to use health care services less frequently than non-newcomers [2], although results related to their use of primary care are mixed [3]. Research and service planning attention are increasingly being devoted to newcomer health, but the needs of newcomers with disabilities remain largely unknown.

While newcomers with disabilities have been underrepresented in research, newcomers with intellectual and developmental disabilities (IDD), such as Down syndrome or autism are even less understood. Recent Canadian prevalence studies have suggested that IDD occurs in approximately 0.5% to 1% of the adult population [4–6]. It is important to focus on newcomers with IDD because IDD is associated with greater morbidity and individuals with IDD experience challenges accessing timely and appropriate health care [1,2]. Some of their difficulties may be related to communication, social skills and problem-solving deficits, which can affect their health issues as well as how they access care [7–9].

Planning that considers the needs of newcomers with IDD requires knowledge of the size of the group. In accordance with the healthy immigrant effect that suggests that many health conditions are less prevalent among newcomers, we expected a lower prevalence of IDD among newcomers than native born persons. This effect is partially attributable to country’s newcomer selection policies that often preclude those with disabilities from being admitted. In Canada this policy stems from the excessive demand clause of the Immigration and Refugee Protection Act (IRPA)[10] that is used to justify the rejection of some newcomers whose expected use of health or social services would likely exceed average Canadian per capita costs for five years. We refer to the group of newcomers that is affected by this policy as ‘screened’ newcomers. However, for some immigrants the decision about whether they are eligible for admission does not depend on their health and social service utilization. Specifically, a proportion of newcomers are typically exempt from this clause, which makes them eligible to become permanent residents irrespective of their health and social service use. We term this group of newcomers as ‘not screened’. Although the excessive demand clause prevents many screened newcomers from arriving or remaining in countries like Canada, the precise prevalence rate is not known. It is difficult to fill these knowledge gaps because population-level data are rarely available on either newcomers or people with IDD. Consequently, despite the policy debates and/or media attention on this issue in several countries (e.g. Canada, Australia, New Zealand, USA) [7–9,11,12], the absence of knowledge about the size of the population of newcomers with IDD and their demographic or health care use profiles has impeded the development of polices to support this group.

Although no individual level studies have compared the prevalence of IDD between adult first generation newcomers and non-newcomers, we found three studies that used convenience samples to report on prevalence. An ecological study showed that in Haute-Garonne, France the prevalence of IDD was higher in areas with a higher concentration of immigrants [13]. Conversely, a study in Leicestershire, United Kingdom on persons over 50 years of age showed a lower prevalence of IDD among South Asian first-generation immigrants compared to White individuals whose immigrant status was not stated [14]. Similarly, a US study observed that a group comprised both of children born outside of the US and US-born children with immigrant parents had a lower prevalence of developmental delay and learning disability than US-born children with US-born parents [15].

To complement the few studies on prevalence of IDD among newcomers [13–15], we found two papers on health and health service use of newcomers with IDD [16,17]; both provided case studies on immigrant families that include a member with a disability. These case studies noted an absence of services targeted to newcomers with IDD. This may be because although resettlement services are versed in mental health and addictions support, they are not versed in IDD support. At the same time, disability services are not focused on newcomer issues. Both studies [16,17] also reported the challenge of the family members not being included in their adult child’s health treatment. This is in contrast to what they were used to in their country of origin where families of adults with IDD were more involved. Challenges accessing appropriate services for newcomers with IDD can be compounded by the individuals’ deficits associated with their IDD, and limited English proficiency [16,17]. Given the challenges with using existing services, one of the case studies [16] noted a need to develop innovative services to support the health needs of newcomers with IDD, as well as their families. However, targeting services to this group requires knowledge of how many newcomers have IDD, what their health issues are, and how these needs compare to other newcomers. This study begins to fill this gap by providing information on newcomers with IDD in Ontario, Canada.

The first objective is to compare the prevalence of IDD among newcomers versus non-newcomers, overall and by age group. We hypothesize that the prevalence of IDD would be lower for newcomers overall because it is hard for those with IDD to move and start a new life in Canada, and because some newcomers would not be permitted to enter due to the excessive demand clause of IRPA described above. We expect this to be observed for all age groups.

Additionally, we explored the prevalence of IDD for newcomer groups with different eligibility criteria (screened, not screened) and with different lengths of time since immigration to Canada (arrived in Canada more than five years ago, arrived more recently) to the prevalence of IDD among non-newcomers.

The second objective is to compare the health status and health service use of newcomers with IDD to newcomers without IDD. We hypothesize that newcomers who have IDD will have more comorbidities and service utilization than other newcomers, which is consistent with the profile of people with IDD in the general population.

There is a unique opportunity to study this topic in Ontario, Canada due to the ability to link health service utilization data across health sectors, and because recent data partnerships permit the linkage and study of population data on adults with IDD and on newcomers.

## Methods

### Setting

This population-based retrospective cohort study was conducted in Ontario, Canada using linked health and social services administrative data, including data on newcomers to Ontario obtained from Immigration, Refugees and Citizenship Canada (IRCC). In 2016 newcomers represented 21.9% of Canada’s total population [18]. The province of Ontario has a single-payer, universal coverage system for physician and hospital services that allows for health services data on Ontario residents with health insurance to become accessible for research. Most newcomers become eligible for provincial health services within the first three to six months in Ontario [19]. This study is timely given the current debate and provincial discord concerning whether Canada and her provinces’ commitments to the equality and human rights extend to people with disabilities [20].

### Data sources and sample

We used data sources maintained by the ICES (formerly known as the Institute for Clinical Evaluative Sciences) at Sunnybrook Health Sciences Centre in Toronto, Ontario. ICES is a non-profit, independent organization that reports on the health and health care utilization of Ontario residents. Data stored at ICES were in an anonymized format before they were accessed by the authors. All datasets were linked using unique encoded identifiers and analyzed at the ICES. The use of data in this project was authorized under section 45 of Ontario’s Personal Health Information Protection Act, which does not require review by a Research Ethics Board. As a result, an ethical review of these anonymized datasets was not required by the Research Ethics Board of the Sunnybrook Health Sciences Centre even though consent was not obtained.

The following databases provided the study variables: the Registered Persons Database; the Ontario Health Insurance Plan (OHIP) database (primary care physician visits); the National Ambulatory Care Reporting System (emergency department [ED] visits); the Canadian Institute for Health Information Discharge Abstract Database (CIHI-DAD, general hospitalizations); the Ontario Mental Health Reporting System (psychiatric hospitalizations); and the Ontario Cancer Registry (for prevalence of cancer algorithm). ICES databases use standardized diagnostic codes from the International Statistical Classification of Diseases and Related Health Problems, and the Diagnostic and Statistical Manual of Mental Disorders to identify individuals with specific conditions from their hospital abstract records and billings of physician provided services.

#### Newcomers

Information on newcomers was obtained through the IRCC database (IRCC-PRD 2012 extract), which identifies newcomers who declared an intention to settle and then did settle in the province of Ontario after 1985; they composed the sample. We used the term newcomers to refer to both immigrants (individuals who are admitted based on his or her ability to become economically established and individuals who are sponsored by family members to come to Canada by a Canadian citizen or permanent resident) and refugees (individuals who fear persecution and who requires protection because they are unwilling or unable to return to their home country) [21]. Newcomers were broken into two groups. The first group of ‘not screened’ newcomers are eligible to become permanent residents irrespective of their health and social service use. This includes all refugees and a subset of immigrants admitted in the family reunification class (i.e., close family class)(S1 Table)[22]. The other newcomer subgroup is composed of those applying in the economic class and those who are more distant family members of a Canadian citizen or permanent resident. As noted earlier, applications for screened newcomers can be rejected if their anticipated use of health or social services costs is likely to exceed the average individual service costs over five-years [10]. This group is referred to as ‘screened’ newcomers.

#### Intellectual and developmental disabilities

We used an algorithm developed by the Health Care Access Research and Developmental Disabilities (H-CARDD) project to identify adults with IDD aged 19 to 65 years on April 1, 2010. IDD was identified from relevant diagnostic codes recorded in health administrative data since inception of each database (≥2 physician visits or ≥1 ED visit or hospitalization) or in disability income support program documentation. Diagnoses included intellectual disability, fetal alcohol syndrome, autism and other pervasive developmental disorders and chromosomal and autosomal anomalies (e.g. Down syndrome, Fragile X syndrome); a list of diagnostic codes is available (S2, S3 and S4 Tables) [5,23,24].

### Measures

#### Demographic characteristics

The Registered Persons Database provided data on age, sex, and postal codes of our study population. We used the Postal Code Conversion File by Statistics Canada [25] to link individuals’ postal codes to census data to obtain rurality scores according to the Ontario Medical Association's 2008 Rurality Index of Ontario [26] and to determine neighbourhood income quintile of place of residence on April 1, 2010 (index date).

#### Physical and mental health conditions

We examined for the presence of chronic physical conditions prior to the index date using validated algorithms that identifies diabetes [27], hypertension [28], chronic obstructive pulmonary disease [29], and congestive heart failure [30] in CIHI-DAD and OHIP databases. We also examined for a history of malignant conditions using the validated Ontario Cancer Registry [31–33].

We identified individuals with psychotic and non psychotic disorders, individuals with substance use disorders and individuals with both a substance use disorder and a psychotic or non psychotic disorder (co-occurring mental health and substance use disorders), which were determined from physician billing claim codes and ICD-10-CA diagnostic codes (S5 Table) in CIHI-DAD, NACRS and OHIP databases from April 1, 2008 to March 31, 2010.

#### Health service use

All-cause service outcomes included: visits to primary care physicians, visits to psychiatrists, ED visits, and hospital admissions. These were determined from OHIP, NACRS and CIHI-DAD respectively.

### Analysis

Using chi-squared tests, we compared the prevalence of IDD among all newcomers relative to all non-newcomers according to age group, screening status, and time since immigration. We compared sociodemographic characteristics between newcomers with and without IDD using t-tests, and χ^2^ tests. We also calculated standardized differences, for which we used a cut-off of 0.10 to conclude if comparisons were meaningfully different [34,35]. Small cells (<6) were not reported to comply with privacy regulations. We calculated the crude prevalence of chronic physical and mental health conditions, as well as service use, among newcomers with and without IDD. In addition, we calculated age- and sex-adjusted risk ratios (aRR) with 95% confidence intervals (CI) using modified Poisson regression models with a robust variance estimator; aRRs were calculated instead of odds ratios since the latter can be biased estimates for common outcomes (greater than 10%) [36]. Analyses were conducted at ICES using SAS version 9.4 (SAS Institute Inc., Cary, NC).

## Results

### Prevalence of IDD among newcomers and non-newcomers

IDD prevalence was lower in newcomers overall relative to non-newcomers overall, based on the standardized difference 0.10 threshold, and all comparisons were significant based on p-values (p-values<0.0001) although the discussion is driven by standardized differences [Table 1]. When prevalence was examined by age group, the youngest adults (aged 19–25 years) had the highest prevalence of IDD across newcomers and non-newcomers; this prevalence was still lower among newcomers compared to their non-newcomer counterparts. When comparing the prevalence of IDD between non-newcomers and newcomer groups with different eligibility criteria and with different lengths of time since arrival, newcomers not screened at arrival and newcomers who arrived less than five years prior to April 1, 2010 had lower prevalence of IDD based on standardized differences. In comparisons of the subgroups of screened newcomers and subgroups of newcomers arriving more than five years earlier, the standardized differences were just below 0.10 and therefore were not considered meaningful differences.

**Table 1:** Prevalence of intellectual and developmental disabilities (IDD) per 100,000 population among newcomers versus non-newcomers (i) overall, (ii) by age group, and within newcomer groups, (iii) with different eligibility criteria, and (iv) with different lengths of time since immigration to Canada, aged 19–65 years in Ontario, Fiscal year 2010.

	n with IDD	IDD Diagnosis per 100,000 adults	n with IDD	IDD Diagnosis per 100,000 adults	Standardized Differences*,**
	(total group n)	(rate)	(total group n)	(rate)	
	Newcomers		Non-newcomers		
(i) Overall	2,830	171.6	61,804	898.3	0.10
	(n = 1,649,633)		(n = 6,880,196)		
(ii) Age group					
19–25 years	844	479.3	12,931	1,492.1	0.10
	(n = 176,085)		(n = 866,627)		
26–49 years	1,594	144.1	32,784	893.7	0.10
	(n = 1,106,561)		(n = 3,668,346)		
50–65 years	392	106.8	16,089	686.0	0.09
	(n = 366,987)		(n = 2,345,223)		
(iii) Screened status			61,804 (n = 6,880,196)	898.3	
Newcomers not screened at arrival	1,447	148.8			0.10
	(n = 972,425)				
Newcomers screened at arrival	1,383	204.2			0.09
	(n = 677,208)				
(iv) Time since immigration			61,804 (n = 6,880,196)	898.3	
0–5 years since immigration	213	57.7			0.12
	(n = 369,443)				
More than 5 years since immigration	2,617	204.4			0.09
	(n = 1,280,190)				

*all p-values <0.0001

**Values <0.1 indicates negligible differences between groups

### Profiles of newcomers with and without IDD

#### Demographic characteristics

Among newcomers, compared to those without IDD (n = 1,646,803), adults with IDD (n = 2,830) were more commonly younger (19–25 years: 29.8% vs. 10.6%), male (52.7% vs. 48.2%), and living in neighbourhoods in the lowest income quintile (31.7% vs. 27.7%) [Table 2]. Newcomers with IDD were also more likely to be admitted as refugees (20.5% vs. 16.0%) and less likely to be admitted in the economic class (37.1% vs. 49.3%) compared to newcomers without IDD.

**Table 2:** Sociodemographic and immigration characteristics of newcomers with and without intellectual and developmental disabilities (IDD), for adults aged 19–65 in Ontario, FY 2010.

		Newcomers without IDD n = 1,646,803	Newcomers with IDD n = 2,830	Standardized Differences*
		n (%)	n (%)	
Age group	19 to 25 years	175,241 (10.6)	844 (29.8)	0.49
	26 to 49 years	1,104,967 (67.1)	1,594 (56.3)	0.22
	50 to 65 years	366,595 (22.3)	392 (13.9)	0.22
Sex	Female	853,339 (51.8)	1,339 (47.3)	0.09
Admission Class	Economic	812,441 (49.3)	1,050 (37.1)	0.25
	Family reunification-child/spouse/common law/conjugal	412,497 (25.0)	803 (28.4)	0.08
	Family reunification-other (not included above)	158,537 (9.6)	397 (14.0)	0.14
	Refugee	263,328 (16.0)	580 (20.5)	0.12
Immigrated within 5 years	Yes	369,230 (22.4)	213 (7.5)	0.43
Neighbourhood income quintile	Missing	3,457 (0.2)	17 (0.6)	0.06
	1 (lowest income)	455,805 (27.7)	896 (31.7)	0.09
	2	371,136 (22.5)	669 (23.6)	0.03
	3	331,429 (20.1)	499 (17.6)	0.06
	4	290,694 (17.7)	441 (15.6)	0.06
	5 (highest income)	194,282 (11.8)	308 (10.9)	0.03
Residing in urban vs rural areas	Rurality Index of Ontario Category 0–9	1,627,723 (98.8)	2,788 (98.5)	0.03
Disability income support	Receiving Ontario Disability Support Program (ODSP)	30,318 (1.8)	1,891 (66.8)	
Morbidity (Resource Utilization Band Category)	No use (0)	249,568 (15.2)	173 (6.1)	0.30
	Low (1 to 3)	1,155,665 (70.2)	2,017 (71.3)	0.02
	High (4 to 5)	241,570 (14.7)	640 (22.6)	0.21

*all p-values <0.0001

#### Physical and mental health profiles

Compared to newcomers without IDD, newcomers with IDD had a higher age- and sex-adjusted prevalence of all the physical and mental health conditions examined [Table 3]. The greatest differences were for psychotic disorders (aRR: 23.26, 95%CI: 21.22–25.50), co-occurring mental health and substance use (aRR: 6.11, 95%CI:5.12–7.30), substance use (aRR: 3.26, 95%CI:2.77–3.84), congestive heart failure (aRR: 2.74, 95%CI:1.55–4.82), and chronic obstructive pulmonary disease (COPD) (aRR: 2.11, 95%CI:1.68–2.66).

**Table 3:** Adjusted prevalence/risk ratios for physical/mental health profiles and health service use of newcomers with and without intellectual and developmental disabilities (IDD), for adults aged 19–65 in Ontario, FY 2010.

	Newcomers without IDD n = 1,646,803	Newcomers with IDD n = 2,830	Newcomer with IDD vs. Newcomer without IDD (REF)
	n (%)	n (%)	Adjusted^Ɨ^ prevalence ratio/risk ratio (95%CI)
**Physical and mental health conditions (prevalence ratios)**			
Diabetes	119,768 (7.3)	280 (9.9)	1.97 (1.77–2.20)
Hypertension	218,783 (13.3)	363 (12.8)	1.42 (1.29–1.55)
Chronic obstructive pulmonary disease	28,343 (1.7)	67 (2.4)	2.11 (1.68–2.66)
Congestive heart failure*	4,243 (0.5)	12 (1.3)	2.74 (1.55–4.82)
Cancer	21,355 (1.3)	44 (1.6)	1.68 (1.26–2.25)
Asthma	101,561 (6.2)	315 (11.1)	1.91 (1.72–2.12)
Non-psychotic disorders	331,320 (20.1)	1,227 (43.4)	2.31 (2.21–2.41)
Psychotic disorders	9,954 (0.6)	410 (14.5)	23.26 (21.22–25.50)
Substance use disorders	23,297 (1.4)	140 (4.9)	3.26 (2.77–3.84)
Co-occurring mental health and substance use disorders	10,672 (0.6)	119 (4.2)	6.11 (5.12–7.30)
**Health service use (risk ratios)**			
Visits to primary care physicians	1,223,792 (74.3)	2,278 (80.5)	1.12 (1.10–1.14)
Visits to psychiatrists	35,987 (2.2)	532 (18.8)	9.32 (8.62–10.08)
Visits to other (non-psychiatrist) specialist physicians	583,527 (35.4)	1,127 (39.8)	1.24 (1.18–1.29)
Emergency department (ED) visits (any)	233,706 (14.2)	695 (24.6)	1.72 (1.61–1.83)
ED visits (5+ visits)	6,141 (0.4)	63 (2.2)	5.92 (4.63–7.57)
Hospital admissions (any)	65,275 (4.0)	145 (5.1)	1.25 (1.06–1.46)
Hospital admissions (2+ admissions)	6,536 (0.4)	25 (0.9)	2.47 (1.67–3.66)

^Ɨ^ Prevalence/risk ratios were derived from modified Poisson regression models adjusted for age and sex

*Frequency counts based on adults age 40 and above

#### Health service use

After adjusting for age and sex, compared to newcomers without IDD, newcomers with IDD were more likely to visit primary care physicians, psychiatrists, and other specialist physicians and to have any ED visits, five or more ED visits, any hospital admissions, and two or more hospital admissions [Table 3]. The most marked differences were that newcomers with IDD were more likely to make at least one psychiatry visit (aRR: 9.32, 95%CI:8.62–10.08), make five or more ED visits within a year (aRR: 5.92, 95%CI:4.63–7.57), and have 2 or more hospitalizations (aRR: 2.47, 95%CI:1.67–3.66).

## Discussion

This is the first study to examine the prevalence of IDD among first generation adult newcomers. The prevalence of IDD was significantly lower among newcomers than non-newcomers in Ontario, Canada. Among newcomers, those with IDD had a higher prevalence of a range of physical and mental health conditions, with marked differences in rates of psychotic and concurrent disorders. Moreover, newcomers with IDD were also significantly more likely to use psychiatry services and have frequent ED visits and hospitalizations than newcomers without IDD.

As noted above, the overall lower IDD prevalence among newcomers may be due to a combination of newcomer self-selection and/or Canadian government selection policies that lead to healthier newcomers (i.e., without IDD) being admitted to Canada, which are also two factors that have been used to explain the healthy immigrant effect. Although rates of IDD were similar for both screened and not screened newcomer groups, rates were lower among newcomers who were not screened at arrival. Since this group is composed of refugees and family members of permanent residents who are typically less physically and financially ‘fit’ than other newcomers [21], these groups may have fewer resources and worse health than newcomers in the screened group that makes arriving in Canada more difficult. One possibility is that newcomers who are not screened are more likely than screened newcomers to come from English speaking countries with service systems that resemble Canada’s health and social services, thereby increasing the likelihood that they use these services upon arrival and that their IDD diagnosis is identified in health and social service databases.

The higher medical and psychiatric comorbidity in people with IDD is consistent with research findings from Ontario and elsewhere on the broader IDD population [37–39]. This difference may be driven in part by unusually low rates of disorders and service use in the comparator group of newcomers without IDD. Lower rates of disorders and service use are consistent with the ‘Healthy Immigrant Effect’ [40–42], although the health advantage normally observed among newcomers may not apply in the same way to those with IDD, although comparing newcomers and non-newcomers with IDD is beyond the scope of this study [16]. However, the rates of psychotic disorders reported for the IDD newcomer group reported in this study are noticeably higher than what is reported for adults with IDD generally. While it is possible that some of this relates to the impact of pre-migration and resettlement stressors, it is also possible that psychotic disorders are being misdiagnosed, at least in some newcomers [43–45]. Diagnosing psychotic disorders can be difficult even in adults with IDD who do not have cultural or language barriers, especially when they have limited verbal ability [46]. On occasion behaviours that appear to be psychotic in nature actually reflect a person’s response to stress and/or being in unfamiliar circumstances [47], and may be better captured by a different diagnosis. For example, difficulties diagnosing psychotic illness in people with autism have been noted [48]. Specifically, it has been suggested that autism may be misdiagnosed as schizophrenia [49] and case studies have shown this can happen specifically in newcomers with IDD [50]. More in depth research is warranted on the prevalence of psychotic disorder, and the challenges with diagnosing them in newcomers with limited English as well as verbal ability.

In terms of health care use, newcomers with IDD were more likely to use psychiatric services and be hospitalized. The higher rates of psychiatrist visits may indicate an ability to access psychiatry at a similar or higher rate to others. However, the crude measures reported here do not provide information on the quantity or quality of such encounters. The quality of these services may be a particular issue for newcomers. Due to their unique cultural and resettlement experiences, they may struggle more than non-newcomers to find a psychiatrist they consider helpful. Few psychiatrists specialize in treating adults with IDD in Canada, so finding a psychiatrist with expertise in newcomers and IDD is highly unlikely [51,52]. Alternatively, newcomers with IDD may choose to delay help-seeking due to fears that they will experience even greater discrimination due to having multiple disadvantaged statuses and/or fears they will be given instructions for follow up care (e.g., medications) without explanation or support [53]. If accessing health services is delayed (for any reason), when services are finally accessed, an individual’s acuity may be higher, potentially leading to greater use of ED visits, admissions and repeat ED and hospital visits [54]. Longitudinal studies would help elucidate these help seeking patterns over time [55].

Newcomers with IDD are more likely to present with health issues than other newcomers once in Canada. These results emphasize the need for newcomers with IDD and their families to be able to access appropriate supports upon arrival. These additional supports could support the health–and particularly the mental health–of newcomers with IDD. For example, these may include increasing the availability of culture-specific interpreter services at health care settings, or increasing access to psychiatrists familiar with particular cultural experiences, but can also include linkages to disability related supports. The recognition of IDD and provision of appropriate supports to newcomers can contribute to health and wellbeing, driven by success in school, employment, and meaningful daytime activities.

We need more research to explore how health care and social services can meet the needs of newcomers with IDD. For example, supporting the health of newcomers with IDD requires different practices than others with IDD, such as providing interpreter services and culturally sensitive explanations of health issues related to IDD and its comorbidities. Training health care providers on issues unique to newcomers, and increasing newcomers and immigration service workers knowledge of Canadian disability accommodation rights and laws are also priorities [56].

### Limitations

There are a number of factors which may contribute to rate of IDD reported in this study. First, our definition of IDD is based on Ontario’s Services and Supports to Promote the Social Inclusion of Persons with Developmental Disabilities Act, 2008, which is broader than the definition of ID only yet considerably narrower than how IDD is defined in US [57]. For example, unlike the US, Ontario does not include learning disabilities, ADHD, or cerebral palsy in its definition. Second, although our cohort was created by merging health and disability support information (an improvement over using health data alone), the literature clearly documents that these kinds of data do not completely capture the IDD population and that information from other sections such as educational or clinical records will contribute unique cases not identified by administrative data [23]. Third, our age range was restricted to individuals aged 19–65. Other research [6,58,59] has shown that rates for children, youth, and younger adults tend to be 2–3 times higher than the rates for adults.

In addition, there may be other factors specifically leading to the underestimation of IDD among newcomers in the current study. IDD is generally diagnosed early in life, and most newcomers will not have ready access to their childhood health records (an important source of information for the diagnostic algorithm used in our study). This possibility is supported by our finding that the highest rates of IDD occur among the youngest newcomers, in other words those with a greater chance of earlier contact with the Ontario health care system. Nevertheless, the fact that the prevalence for the younger newcomers is still lower than for the younger non-newcomers suggests the influence of other factors.

It may be that certain types of IDD such as autism are more difficult to diagnose among newcomers, due to language, poor familiarity with the healthcare and educational systems, witnessing traumatic events, and the absence of screening tools tailored to particular newcomer or ethnic groups [54,60,61]. Since the data for this study are from 2009/10, and the composition of the newcomer population in Canada has changed since that time [62,63], studies with more recent data will be critical. Furthermore, this study did not include newcomers who entered Ontario from a different province, refugee claimants who have not been accepted or are in the process of appealing, and other temporary residents, workers, visitors, or “non-status” residents. In terms of physical and mental health conditions, the comorbidities we examined included conditions that are relevant to people with IDD and are identifiable using Ontario's administrative data sources. However, these algorithms have not been validated specifically for those with IDD. Additionally, we were able to use these algorithms to illustrate the likelihood of various conditions diagnosed once living in Ontario, but it is not known whether these health conditions developed before or after arrival.

### Future research

Future work should explore how much newcomers with IDD utilize healthcare services relative to other people with IDD, and the timing of the emergence of health issues relative to IDD in health records. Future studies on newcomers with IDD should also study young children separately from adults who immigrate at an older age. This is because it has been shown that for newcomers without IDD, the experience of those who arrive as children and as adults are different [64]. This is likely also true for those with IDD, and these differences may even be amplified because integration and learning of culture happens in school. Younger children can participate in special education on a full-time basis and have a day-to-day structure which helps them and their family. Newcomers who are older at arrival may not have the same opportunities for supported education or employment. This study focused on adults because a critical administrative database used to identify the cohort with IDD (disability income support) was limited to adults between the ages of 18 to 64 when the cohort was created in fiscal year 2009. No comparable population-based administrative data were available for either younger or older adults. It would also be important to explore whether the high rates of mental health and addiction issues and associated service use seen in newcomers with IDD are also evident in newcomers with other types of disabilities. Examining a more recent cohort of people with IDD, and people with different types of IDD, would also be advantageous to reflect more recent newcomer patterns.

Although many more questions remain, there has been a total knowledge gap on the prevalence of IDD among Canadian newcomers and on their health care use. This gap has persisted despite significant advocacy and media attention being directed toward the appropriateness of restricting newcomers with disabilities from coming to Canada. These early data allow us to begin to address pertinent questions, and open up avenues to discuss future research priorities [65].

## Conclusion

Given the knowledge gap related to newcomers and disabilities, particularly IDD, this population-based study is an early step toward building an evidence base. Present findings suggest a much lower prevalence of IDD among newcomers compared to Canadian-born persons. Self-selection may be a contributor to these findings, however, it was not possible to estimate the effect of Canadian selection policies on the exclusion of newcomer applicants with IDD.

Newcomers with IDD would likely benefit from additional supports being made available shortly after their arrival since adult newcomers living in Canada experience more mental health conditions, greater psychiatry visits and greater hospital use than other adult newcomers in Canada.

## Supporting information

S1 Table Classification of newcomers who are ‘not screened’ and ‘screened’ at arrival. (DOCX)

S2 Table Diagnostic codes used to identify individuals with developmental disabilities in the administrative health data. (DOCX)

S3 Table Developmental disabilities and related codes included in the International Classification of Diseases, 9th and 10th editions. (DOCX)

S4 Table ICD-9 codes used to identify individuals with developmental disabilities in the Ontario Disability Support Program (ODSP) database. (DOCX)

S5 Table Codes for psychotic, non-psychotic, substance use disorders, and co-occurring mental health and substance use disorders. (DOCX)

## References

[1] Vang Z, Sigouin J, Flenon A, Gagnon A. The healthy immigrant effect in Canada: A systematic review. PCLC. 2015;3(1):1–41. 10.1080/13557858.2016.1246518 27809589

[2] Derr AS. Mental health service use among immigrants in the United States: A systematic review. Psychiatr Serv. 2017;67(3):265–74. 10.1176/appi.ps.201500004 PMC5122453 26695493

[3] Uiters E, Devillé W, Foets M, Spreeuwenberg P, Groenewegen PP. Differences between immigrant and non-immigrant groups in the use of primary medical care; A systematic review. BMC Health Serv Res. 2009;9:1–10. 10.1186/1472-6963-9-1 19426567 PMC2689181

[4] Morris S, Fawcett G, Brisebois L, Hughes J. A demographic, employment and income profile of Canadians with disabilities aged 15 years and over, 2017 [Internet]. Ottawa; 2018. Available from: https://www150.statcan.gc.ca/n1/pub/89-654-x/89-654-x2018002-eng.pdf

[5] Lin E, Balogh R, Cobigo V, Ouellette-Kuntz H, Wilton AS, Lunsky Y. Using administrative health data to identify individuals with intellectual and developmental disabilities: A comparison of algorithms. J Intellect Disabil Res. 2013;57(5):462–77. 10.1111/jir.12002 23116328

[6] Oullette-Kuntz H, Shooshtari S, Temple B, Brownell M, Burchill C, Yu CT, et al Estimating administrative prevalence of intellectual disabilities in Manitoba. J Devlop Disabi. 2010;15(3):69–80.

[7] Stayner G. Woman with intellectual disabilities faces deportation after mother’s visa rejection. ABC News [Internet]. 2017 Sep 7; Available from: http://mobile.abc.net.au/news/2017-09-07/woman-with-intellectual-disabilities-faces-deportation-to-india/8879358?pfmredir=sm

[8] Balint R. Australia has kept disabled migrant children out for decades–it’s time we gave them protection instead. The Conversation [Internet]. 2017 Mar 1; Available from: http://theconversation.com/australia-has-kept-disabled-migrant-children-out-for-decades-its-time-we-gave-them-protection-instead-73677

[9] Leemans D. My son’s autism meant he was refused New Zealand residency–so we’re leaving. The Guardian [Internet]. 2016 Feb 17; Available from: https://www.theguardian.com/commentisfree/2016/feb/17/my-stepson-autism-new-zealand-refused-residency-asd

[10] Government of Canada. Excessive demand on health and social services [Internet]. 2017 [cited 2017 Oct 2]. Available from: http://www.cic.gc.ca/english/resources/tools/medic/admiss/excessive.asp

[11] Davis MR. Trump administration seeks to bar immigrants with disabilities. Disability Scoop [Internet]. 2018 4 30; Available from: https://www.disabilityscoop.com/2018/04/30/trump-bar-immigrants-disabilities/25032/

[12] Smith S. Changes coming to Canada’s medical inadmissibility rules. CIC News [Internet]. 2017 11 23; Available from: https://www.cicnews.com/2017/11/changes-coming-to-canadas-medical-inadmissibility-rules-119878.html#gs.2kR9sF4

[13] Delobel-ayoub M, Ehlinger V, Klapouszczak D. Socioeconomic disparities and prevalence of autism spectrum disorders and intellectual disability. PLoS One. 2015;10(111):1–13. 10.1371/journal.pone.0141964 PMC4635003 26540408

[14] Mcgrother CW, Bhaumik S, Thorp CF, Watson JM, Taub NA, Healthcare R, et al Prevalence, morbidity and service need among South Asian and white adults with intellectual disability in Leicestershire, UK. J Intellect Disabil Res. 2002;46(4):299–309. 12000581 10.1046/j.1365-2788.2002.00391.x

[15] Singh GK, Yu SM, Kogan MD. Health, chronic conditions, and behavioral risk disparities among U.S. immigrant children and adolescents. Public Health Rep. 2013;128(6):468–79. 10.1177/003335491312800606 PMC3804090 24179258

[16] King J, Edwards N, Correa-velez I, Darracott R, Fordyce M. Restrictive practices on refugees in Australia with intellectual disability and challenging behaviours: a family’s story. Adv Ment Heal Intellect Disabil. 2016;10(4):222–32.

[17] Bogenshutz M. “We find a way”: Challenges and facilitators for health care access among immigrants and refugees with intellectual and developmental disabilities. Med Care. 2014;52(10):64–70. 10.1097/MLR.0000000000000140 25215921

[18] Statistics Canada. Immigration and ethnocultural diversity: Key results from the 2016 Census [Internet]. 2017 [cited 2019 Apr 1]. Available from: https://www150.statcan.gc.ca/n1/daily-quotidien/171025/dq171025b-eng.htm

[19] Ontario Ministry of Health and Long-Term Care. OHIP coverage waiting period [Internet]. 2011 [cited 2017 Oct 6]. Available from: http://www.health.gov.on.ca/en/public/publications/ohip/ohip_waiting_pd.aspx

[20] Oliphant R. Building an inclusive Canada: Bringing Immigration and Refuge Protection Act in step with modern values [Internet]. Ottawa; 2017 [cited 2018 Sep 18]. Available from: http://www.ourcommons.ca/Content/Committee/421/CIMM/Reports/RP9322080/cimmrp15/cimmrp15-e.pdf

[21] Durbin A, Lin E, Moineddin R, Steele LS, Glazier RH. Use of mental health care for nonpsychotic conditions by immigrants in different admission classes and by refugees in Ontario, Canada. Open Med. 2014;8(4):e136–146. 25426182 PMC4242791

[22] Government of Canada. Immigration and Refugee Protection Act (S.C. 2001, c. 27) [Internet]. 2017 [cited 2017 Sep 18]. Available from: http://laws-lois.justice.gc.ca/eng/acts/I-2.5/section-38.html

[23] Lin E, Balogh R, Isaacs B, Ouellette-Kuntz H, Selick A, Wilton AS, et al Strengths and limitations of health and disability support administrative databases for population-based health research in intellectual and developmental disabilities. J Policy Pract Intellect Disabil. 2014;11(4):235–44.

[24] Lunsky Y, Klein-Geltink JE, Yates EA. Atlas on the primary care of adults with developmental disabilities in Ontario. Toronto; 2013. 10.12927/hcq.2014.24026 25591603

[25] Wilkins R. PCCF+ version 5C user’s guide: Automated geographic coding based on the statistics Canada postal code conversion files. Ottawa; 2008.

[26] Kralj B. Measuring rurality—RIO2008_BASIC: Methodology and results. 2009.

[27] Hux J, Ivis F, Flintoft V, Bica A. Diabetes in Ontario: Determination of prevalence and incidence using a validated administrative data algorithm. Diabetes Care. 2002;25:512–6. 10.2337/diacare.25.3.512 11874939

[28] Tu K, Campbell N, Chen Z, Cauch-Dudek K, McAlister F. Accuracy of administrative databases in identifying patients with hypertension. Open Med. 2007;1:e18–26. 20101286 PMC2801913

[29] Gershon A, Wang C, Guan J, Vasilevska-Ristovska J, Cicutto L, To T. Identifying individuals with physcian diagnosed COPD in health administrative databases. COPD. 2009;6:388–94. 19863368 10.1080/15412550903140865

[30] Schultz S, Rothwell D, Chen Z, Tu K. Identifying cases of congestive heart failure from administrative data: A validation study using primary care patient records. Chronic Dis Inj Can. 2013;33(3):160–6. 23735455

[31] Hall S, Shulze K, Groome P, Mackillop W, Holowaty E. Using cancer registry data for survival studies: the example of the Ontario Cancer Registry. J Clin Epidemiol. 2006;59(1):67–76. 10.1016/j.jclinepi.2005.05.001 16360563

[32] Acuna S, Fernandes K, Daly C, Hicks L, Sutradhar R, Kim S, et al Cancer Mortality Among Recipients of Solid-Organ Transplantation in Ontario, Canada. JAMA Oncol. 2016;2(4):463–9. 10.1001/jamaoncol.2015.5137 26746479

[33] James P, Hegagi M, Antonova L, Tinmouth J, Heitman S, Simone C, et al Regional differences in use of endoscopic ultrasonography in Ontario: a population-based retrospective cohort study. C Open. 2017;5(2):E437–443. 10.9778/cmajo.20160153 PMC5498178 28600449

[34] Austin PC. Using the standardized difference to compare the prevalence of a binary variable between two groups in observational research. Commun Stat Simul Comput. 2009;38(6):1228–34.

[35] Flury BK, Riedwyl H. Standard distance in univariate and multivariate analysis. Am Stat. 1986;40(3):249–51.

[36] McNutt LA, Wu C, Xue X, Hafner JP. Estimating the relative risk in cohort studies and clinical trials of common outcomes. Am J Epidemiol. 2003;157(10):940–3. 10.1093/aje/kwg074 12746247

[37] Lin E, Balogh R, Mcgarry C, Selick A, Dobranowski K, Wilton AS, et al Substance-related and addictive disorders among adults with intellectual and developmental disabilities (IDD): an Ontario population cohort study. BMJ Open. 2016;6:1–7. 10.1136/bmjopen-2016-011638 PMC5020882 27591020

[38] Cooper S-A, McLean G, Guthrie B, McConnachie A, Mercer S, Sullivan F, et al Multiple physical and mental health comorbidity in adults with intellectual disabilities: population-based cross-sectional analysis. BMC Fam Pract. 2015;16(1):110. 26310664 10.1186/s12875-015-0329-3 PMC4551707

[39] Emerson E, Hatton C. Health inequalities and people with intellectual disabilities Cambridge: Cambridge University Press; 2014.

[40] Durbin A, Moineddin R, Lin E, Steele LS, Glazier RH. Mental health service use by recent immigrants from different world regions and by non-immigrants in Ontario, Canada: A cross-sectional study. BMC Health Serv Res. 2015;15(1):1–15. 26290068 10.1186/s12913-015-0995-9 PMC4546085

[41] Newbold KB, Danforth J. Health status and Canada’s immigrant population. Soc Sci Med. 2003;57(10):1981–95. 14499520 10.1016/s0277-9536(03)00064-9

[42] McDonald JT, Kennedy S. Insights into the “healthy immigrant effect”: Health status and health service use of immigrants to Canada. Soc Sci Med. 2004;59(8):1613–27. 10.1016/j.socscimed.2004.02.004 15279920

[43] Anderson K, Cheng J, Susser E, McKenzie K, Kurdyak P. Incidence of psychotic disorders among first-generation immigrants and refugees in Ontario. CMAJ. 2015;187(9):279–86. 10.1503/cmaj.141420 PMC4467956 25964387

[44] Haasen C, Yagdiran O, Mass R, Krausz M. Potential for misdiagnosis among Turkish migrants with psychotic disorders: A clinical controlled study in Germany. Acta Psychiatr Scand. 2000;101(2):125–9. 10706012 10.1034/j.1600-0447.2000.90065.x

[45] Schwartz RC. Racial disparities in psychotic disorder diagnosis: A review of empirical literature. World J Psychiatry. 2014;4(4):133 10.5498/wjp.v4.i4.133 25540728 PMC4274585

[46] Cooper S, Smiley E, Morrison J, Allan L, Williamson A, Finlayson J, et al Psychosis and adults with intellectual disabilities. Prevalence, incidence, and related factors. Soc Psychiatry Psychiatr Epidemiol. 2007;42(7):530–6. 10.1007/s00127-007-0197-9 17502974

[47] Khanlou N, Mustafa N. Stressors and barriers to services for immigrant fathers raising children with developmental disabilities. Int J Ment Health Addict. 2015;13(6):659–74. 10.1007/s11469-015-9584-8 26568705 PMC4639580

[48] Lai M, Baron-Cohen S. Identifying the lost generation of adults with autism spectrum conditions. Lancet Psychiatry. 2015;2(11):1013–27. 10.1016/S2215-0366(15)00277-1 26544750

[49] Tantam D. Lifelong eccentricity and social isolation. I. Psychiatric, social, and forensic aspects. Br J Psychiatry. 1988;153:777–82. 3273368 10.1192/bjp.153.6.777

[50] Palucka A, Bradley E, Lunsky Y. A case of unrecognized intellectual disability and autism misdiagnosed as schizophrenia: Are there lessons to be learned? Ment Heal Asp Dev Disabil. 2008;11(2):55–60.

[51] Lunsky Y, Garcin N, Morin D, Cobigo V, Bradley E. Mental health services for individuals with intellectual disabilities in Canada: findings from a national survey. J Appl Res Intellect Disabil. 2007;20(5):439–47.

[52] Lunsky Y, Lake J, Balogh R, Weiss J, Morris S. A review of Canadian mental health research on intellectual and developmental disabilities. J Ment Health Res Intellect Disabil. 2013;6(2):106–26.

[53] DeCoux Hampton M. The role of treatment setting and high acuity in the overdiagnosis of schizophrenia in African Americans. Arch Psychiatr Nurs. 2007;21(6):327–35. 10.1016/j.apnu.2007.04.006 18037443

[54] Palucka A, Modi M, Lunsky Y. Changing profiles of individuals with autism spectrum disorder admitted to a specialized inpatient unit. Adv Autism. 2017;3(1):27–33.

[55] Lunsky Y, Tint A, Robinson S, Gordeyko M, Ouellette‐Kuntz H. System‐wide information about family carers of adults with intellectual/developmental disabilities—a scoping review of the literature. J Policy Pract Intellect Disabil. 2014;11(1).

[56] Khanlou N, Haque N, Mustafa N. Access barriers to services by immigrant mothers of children with autism in Canada. Int J Ment Health Addict. International Journal of Mental Health and Addiction; 2017;15(2):239–59. 10.1007/s11469-017-9732-4 28424567 PMC5378730

[57] Government of Ontario. Services and Supports to Promote the Social Inclusion of Persons with Developmental Disabilities Act, 2008. S.O. 2008, c. 14. Available from: http://www.e-laws.gov.on.ca/html/statutes/english/elaws_statutes_08s14_e.htm

[58] Maulik PK, Mascarenhas MN, Mathers CD, Dua T, Saxena S. Prevalence of intellectual disability: a meta-analysis of population-based studies. Res Dev Disabil. 2011;32(2):419–36. 10.1016/j.ridd.2010.12.018 21236634

[59] Boyle CA, Boulet S, Schieve LA, Cohen RA, Blumberg SJ, Yeargin-Allsopp M, et al Trends in the prevalence of developmental disabilities in US children, 1997–2008. Pediatrics. 2011;127(6):1034–42. 10.1542/peds.2010-2989 21606152

[60] Sohn E. Why autism seems to cluster in some immigrant groups [Internet]. 2017 [cited 2017 Dec 29]. Available from: https://spectrumnews.org/features/deep-dive/why-autism-seems-to-cluster-in-some-immigrant-groups/

[61] Bhayana A, Bhayana B. Approach to developmental disabilities in newcomer families. Can Fam Physician. 2018;64(8):567–73. 30108071 PMC6189880

[62] Government of Canada. #WelcomeRefugees: Key figures [Internet]. 2017 [cited 2017 Oct 6]. Available from: http://www.cic.gc.ca/english/refugees/welcome/milestones.asp

[63] Morency J, Malenfant E, MacIsaac S. Immigration and diversity: Population projections for Canada and its Regions, 2011 to 2036. Ottawa; 2017.

[64] Beck A, Corak M, Tienda M. Age at immigration and the adult attainments of child migrants to the united states. Ann Am Acad Pol Soc Sci. 2012;643(1):134–59. 10.1177/0002716212442665 23105147 PMC3478675

[65] Harris K. Liberals to scrap policy that rejects sick, disabled immigrants. Canadian Broadcasting Corporation [Internet]. 2017 11 22; Available from: http://www.cbc.ca/news/politics/hussen-immigration-medical-disability-1.4414274

