# Supplementary material for: Prevalence of intellectual and developmental disabilities among first generation adult newcomers, and the health and health service use of this group: A retrospective cohort study
Source: PLoS One. 2019 Jun 20;14(6):e0215804. doi: 10.1371/journal.pone.0215804 (PMC6586270; doi:10.1371/journal.pone.0215804)
Supplement: S1 Table — (DOCX) [file pone.0215804.s001.docx]

**S1 Table.** Classification of newcomers who are ‘not screened’ and ‘screened’ at arrival

**Not screened newcomers**

- FC3-Son or daughter
- FC1-Spouse
- FCE-Conjugal partner
- FCC-Common law partner
- CR8-Inland: Refugee recognized by the IRB (Principal applicant may include dependants) - To be determined
- CR1-Abroad: Government assistance required
- DR2-Abroad: Dependant of a CR8 (principal applicant determined by the IRB) - Dependant NOT deemed a CR by a visa officer abroad
- CR3-Abroad: Privately sponsored by a group or corporation for 12 months
- CR5-Abroad: Special needs case selected under joint assistance sponsorship (JAS)
- DR1-Inland: Dependant of a CR8 (principal applicant determined by the IRB) - Dependant NOT determined a CR by the IRB
- CR2-Abroad: Dependent of a CR - Sponsored as assisted relative or family class
- CR4-Abroad: Self-Supporting - Government assistance not required
- CRS-Convention refugee abroad sponsored by SAH
- CR6-Inland: Dependant of CR selected abroad (Principal applicant is a resettled refugee)

**Screened newcomers**

- FC4-Parent or grandparent
- FC8-Parent of Canadian citizen
- FC9-Child adopted by a Canadian citizen or a permanent resident
- FC2-Fiancé(e)
- FC5-Orphaned: Brother, sister, nephew, niece, grandchild
- FC6-Inland: Child to be adopted
- FC7-Other relative
- AR3-Parent
- FCH-Family relations - H&C
- DC3-Abroad: Sponsored by group or corporation
- DC1-Abroad: Government assistance required
- RA3-Abroad: Sponsored by a group or corporation for 12 months
- RAG-Country of asylum sponsored by group of five
- DC2- Abroad-Approved IMM1298 or IMM1344 on file as assisted relative or family class
- DC5-Abroad: Designated class - Selected under joint assistance sponsorship (JAS)
- DC4-Abroad: Self-Supporting - Government assistance not required
- RS5-Abroad: Special needs case selected under joint assistance sponsorship (JAS)
- RAS-Country of asylum sponsored by SAH
- RS1-Abroad: Government assistance required
- RAC-Country of asylum with community sponsorship
- PTR-Protected Temporary Resident Class
