# Supplementary material for: Prevalence of intellectual and developmental disabilities among first generation adult newcomers, and the health and health service use of this group: A retrospective cohort study
Source: PLoS One. 2019 Jun 20;14(6):e0215804. doi: 10.1371/journal.pone.0215804 (PMC6586270; doi:10.1371/journal.pone.0215804)
Supplement: S2 Table — (DOCX) [file pone.0215804.s002.docx]

**S2 Table.** Diagnostic codes used to identify individuals with developmental disabilities in the administrative health data

| **Database** | **Year of inception** | **Diagnoses** | **Criteria** |
| --- | --- | --- | --- |
| Discharge Abstract Database | 1988 | Discharges with any diagnosis listed in Exhibit A.2 | In any diagnostic field  For all facilities submitting to DAD, SDS and NACRS  From inception of database to March 31, 2010 |
| Same Day Surgery Database | 1991 |  |  |
| National Ambulatory Care Reporting System | 2002 |  |  |
| Ontario Mental Health Reporting System | 2005 | Q3 = 1  or Q2aa, Q2ab or Q2ac (i.e., Axis I) in 299 to 299.80 or Q2b (i.e., Axis II) in 317 to 319.99 or I11a–I11f = any diagnosis of Qxxx as listed in Exhibit A.2 | For all facilities submitting to OMHRS  From inception of database to March 31, 2010 |
| Ontario Health Insurance Plan | 1991 | 299, 319 | For all providers submitting to OHIP  From June 1991 to March 31, 2010 |
