# Supplementary material for: Prevalence of intellectual and developmental disabilities among first generation adult newcomers, and the health and health service use of this group: A retrospective cohort study
Source: PLoS One. 2019 Jun 20;14(6):e0215804. doi: 10.1371/journal.pone.0215804 (PMC6586270; doi:10.1371/journal.pone.0215804)
Supplement: S3 Table — (DOCX) [file pone.0215804.s003.docx]

**S3 Table.** Developmental disabilities and related codes included in the International Classification of Diseases, 9th and 10th editions

| **Code** | **Label** |
| --- | --- |
| **ICD-9** |  |
| 299–299.99 | Pervasive developmental disorders (e.g., autism) |
| 317–317.99 | Mental retardation |
| 318–318.99 | Mental retardation |
|  |  |
| 319–319.99 | Mental retardation |
|  |  |
| 758.0–758.39 | Chromosomal anomalies for which a developmental disability is typically present |
|  |  |
| 758.5 | Other conditions due to autosomal anomalies |
|  |  |
| 758.8, 758.89 | Other conditions due to chromosome anomalies (do not include 758.81) |
|  |  |
| 758.9 | Conditions due to anomaly of unspecified chromosome |
|  |  |
| 759.5 | Tuberous sclerosis |
|  |  |
| 759.81 | Other and unspecified congenital anomalies: Prader-Willi syndrome |
|  |  |
| 759.821 | Other and unspecified congenital anomalies: de Lange syndrome (include only if 6 digits exist; i.e., do not include 759.82) |
|  |  |
| 759.827 | Other and unspecified congenital anomalies: Seckel syndrome (include only if 6 digits exist) |
|  |  |
| 759.828 | Other and unspecified congenital anomalies: Smith-Lemli-Opitz syndrome (include only if 6 digits exist) |
|  |  |
| 759.83 | Other and unspecified congenital anomalies: Fragile X syndrome |
|  |  |
| 759.874 | Other and unspecified congenital anomalies: Beckwith-Wiedemann syndrome (include only if 6 digits exist) |
|  |  |
|  |  |
| 759.875 | Other and unspecified congenital anomalies: Zellweger syndrome (include only if 6 digits exist) |
|  |  |
|  |  |
| 759.89 | Other and unspecified congenital anomalies: other  (e.g.,Menkes disease, Laurence-Moon-Biedl syndrome, Rubinstein-Taybi syndrome) |
|  |  |
| 760.71 | Fetal alcohol syndrome |
| 760.77 | Fetal hydantoin syndrome |
| ICD-10 |  |
| F700 | Mild mental retardation with the statement of no, or minimal, impairment of behaviour |
| F701 | Mild mental retardation, significant impairment of behaviour requiring attention or treatment |
| F708 | Mild mental retardation, other impairments of behaviour |
| F709 | Mild mental retardation without mention of impairment of behaviour |
| F710 | Moderate mental retardation with the statement of no, or minimal, impairment of behaviour |
|  |  |
| F711 | Moderate mental retardation, significant impairment of behaviour requiring attention or treatment |
|  |  |
| F718 | Moderate mental retardation, other impairments of behaviour |
| F719 | Moderate mental retardation without mention of impairment of behaviour |
| F720 | Severe mental retardation with the statement of no, or minimal, impairment of behaviour |
| F721 | Severe mental retardation, significant impairment of behaviour requiring attention or treatment |
| F728 | Severe mental retardation, other impairments of behaviour |
| F729 | Severe mental retardation without mention of impairment of behaviour |
| F730 | Profound mental retardation with the statement of no, or minimal, impairment of behaviour |
| F731 | Profound mental retardation, significant impairment of behaviour requiring attention or treatment |
|  |  |
| F738 | Profound mental retardation, other impairments of behaviour |
|  |  |
| F739 | Profound mental retardation without mention of impairment of behaviour |
|  |  |
| F780 | Other mental retardation with the statement of no, or minimal, impairment of behaviour |
|  |  |
| F781 | Other mental retardation, significant impairment of behaviour requiring attention or treatment |
|  |  |
| F788 | Other mental retardation, other impairments of behaviour |
|  |  |
| F789 | Other mental retardation without mention of impairment of behaviour |
|  |  |
| F790 | Unspecified mental retardation with the statement of no, or minimal, impairment of behaviour |
|  |  |
| F791 | Unspecified mental retardation, significant impairment of behaviour requiring attention or treatment |
|  |  |
| F798 | Unspecified mental retardation, other impairments of behaviour |
|  |  |
| F799 | Unspecified mental retardation without mention of impairment of behaviour |
| F840 | Childhood autism |
| F841 | Atypical autism |
|  |  |
| F843 | Other childhood disintegrative disorder |
|  |  |
| F844 | Overactive disorder associated with mental retardation and stereotyped movements |
| F845 | Asperger’s syndrome |
|  |  |
| F848 | Other pervasive developmental disorders |
| F849 | Pervasive developmental disorder, unspecified |
|  |  |
| Q851 | Tuberous sclerosis |
| Q860 | Fetal alcohol syndrome |
| Q861 | Fetal hydantoin syndrome |
| Q871 | Aarskog, Prader-Willi, deLange, Seckel, etc. |
| Q8723 | Rubinstein-Taybi syndrome (include only if all 5 digits) |
| Q8731 | Sotos syndrome (include only if all 5 digits) |
| Q878 | Other |
| Q900–Q939  except Q926 | All Down syndrome types, cri du chat, etc., except extra marker chromosomes |
|  |  |
| Q971 | Female with more than three X chromosomes |
|  |  |
| Q992 | Fragile X syndrome |
| Q998 | Other specified chromosome abnormalities |
