# Supplementary material for: Prevalence of intellectual and developmental disabilities among first generation adult newcomers, and the health and health service use of this group: A retrospective cohort study
Source: PLoS One. 2019 Jun 20;14(6):e0215804. doi: 10.1371/journal.pone.0215804 (PMC6586270; doi:10.1371/journal.pone.0215804)
Supplement: S4 Table — (DOCX) [file pone.0215804.s004.docx]

**S4 Table.** ICD-9 codes used to identify individuals with developmental disabilities in the Ontario Disability Support Program (ODSP) database

| **ICD-9 Code** | **Description** |
| --- | --- |
| 299 | Pervasive developmental disorders |
| 759 | Other and unspecified congenital anomalies |
| 760 | Fetus or newborn affected by maternal conditions that may be unrelated to present pregnancy |
| 317 | Mild mental retardation |
| 318 | Other specified mental retardation |
| 319 | Unspecified mental retardation |
| 758 | Chromosomal anomalies |
