# Supplementary material for: Prevalence of intellectual and developmental disabilities among first generation adult newcomers, and the health and health service use of this group: A retrospective cohort study
Source: PLoS One. 2019 Jun 20;14(6):e0215804. doi: 10.1371/journal.pone.0215804 (PMC6586270; doi:10.1371/journal.pone.0215804)
Supplement: S5 Table — (DOCX) [file pone.0215804.s005.docx]

**S5 Table.** Codes for psychotic, non-psychotic, substance use disorders, and co-occurring mental health and substance use disorders

| **Category** | **Coding system: Label** | **Codes** |
| --- | --- | --- |
| **Mental Illness: Psychotic**  **Disorders** | **OHIP** | |
|  | Schizophrenia | 295 |
|  | Paranoid states | 297 |
|  | Other psychoses | 298 |
|  | Childhood psychoses (e.g., autism) - 299 | EXCLUDED |
|  | **ICD-9** | |
|  | Schizophrenic disorders | 295 |
|  | Delusional disorders | 297 |
|  | Other organic psychoses | 298 |
|  | **ICD-10** | |
|  | Schizophrenia, schizotypal, delusional, other psychotic, schizoaffective, | F2 |
|  | **DSM-IV** | |
|  | Psychotic disorders due to medical conditions | 293.81 , 293.82  (Note: 298.83 is coded under ‘Other’) |
|  | Schizophrenia, schizophreniform, schizoaffective | 295 (inclusive) |
|  | Delusional,  shared psychotic disorder | 297 (inclusive) |
|  | Brief and psychotic disorders | 298 (inclusive) |
|  | **OMHRS provision diagnosis (use only if no DSM-IV diagnosis)** | |
|  | DSM: Schizophrenia, other psychotic disorder | Q1E = 1 |
| **Mental Illness : Non-psychotic disorders**  **(non-psychotic, continued)** | **OHIP** | |
|  | Senile/presenile dementia | 290 |
|  | Manic depressive psychosis, involutional melancholia | 296 |
|  | Anxiety neuroses, reactive depression, etc. | 300 |
|  | Personality disorders | 301 |
|  | Sexual deviations | 302 |
|  | Psychosomatic disturbances | 306 |
|  | Habit spasms, tics, stuttering, tension headaches, anorexia nervosa, sleep disorders, enuresis | 307 |
|  | Adjustment reaction | 309 |
|  | Depressive or other non-psychotic disorders, nec | 311 |
|  | Behaviour disorders of childhood and adolescence* | 313 |
|  | Hyperkinetic syndrome of childhood* | 314 |
|  | Specific delays in development (e.g., dyslexia, ..motor retardation) | EXCLUDED |
|  | Mental retardation (319) | EXCLUDED |
|  | **ICD-9** | |
|  | Dementias | 290 |
|  | Other transient disorders (e.g., delirium) | 293 |
|  | Persistent mental illnesses due to other conditions (e.g. amnestic disorders, Alzheimer’s) | 294 |
|  | Episodic mood disorders | 296 |
|  | Anxiety states also including  Dissociative, conversion, and factitious disorders (300.1)  Phobic disorders (300.2)  OCD (300.3)  **Dysthymia (300.4) (note; this is a mood disorder)**  Neurasthenia, derealization, hypochondriasis, somatoform, unspecified non-psychotic (300.5-300.9) | 300 |
|  | Personality disorders | 301 |
|  | Sex and gender-related disorders | 302 |
|  | Psychogenic malfxn from mental factors | 306 |
|  | Specific sx, NEC (e.g., stuttering, eating disorders, tics, etc.) | 307 |
|  | Acute stress reaction | 308 |
|  | Adjustment reaction | 309 |
|  | Non-psychotic conditions due to brain damage | 310 |
|  | Depressive disorder, nec | 311 |
|  | Disturbance of conduct* | 312 |
|  | Disturbance of emotions specific to childhood* | 313 |
|  | Hyperkinetic syndrome of childhood (e.g., ADD)* | 314 |
|  | Specific delays in development (e.g., reading) | EXCLUDED |
|  | Psychic factors associated with diseases classified elsewhere | 316 |
|  | **ICD-10** | |
|  | Dementia (Alzheimer’s, vascular, other diseases, unspecified)  Organic amnestic syndrome  Delirium  Other mental and personality disorders due to brain damage, disease  Unspecified organic disorder | F0 (includes F00-F09) |
|  | Manic, bipolar, depressive disease, cyclothymia | F3 |
|  | Eating disorders, nonorganic sleep, sexual dysfunction, associated with puerperium, etc. | F50, F51, F52, F53 |
|  | Phobias, panic, GAD | F40, F41 |
|  | OCD | F42 |
|  | Stress reaction, PTSD, etc  (includes adjustment disorder – F43.2) | F43 |
|  | Dissociative, somatoform,  Other (neurasthenia, depersonalization) | F44, F45, F46, F48 |
|  | Psychological and behavioural factors associated with disorders of diseases classified elsewhere; abuse of non-dependence-producing substances; unspecified behavioural syndromes | F54, F55, F59 |
|  | Personality disorders, mixed personality disorders, enduring personality change | F60, F61, F62 |
|  | Habit and impulse disorders | F63 |
|  | Gender identity disorders, disorders of sexual preference, disorders associated with sexual development and orientation | F64, F65, F66 |
|  | Other disorders of adult personality and behavior (including Munchhausen’s),  Unspecified | F68, F69 |
|  | Mental retardation (F7)  Disorders of psychological development, scholastic skills, pervasive developmental disorders (F8) | EXCLUDED |
|  | Hyperkinetic, conduct disorders, separation anxiety, attachment disorders, tic disorders, stammering* | F90, F91, F92, F93, F94, F95, F98 |
|  | Mental disorder, NOS | F99 |
|  | **DSM-IV** | |
|  | Mood disorder, NOS | 206.90 |
|  | Dementias | 290 |
|  | Mental conditions due to medical conditions | 293 (but NOT 293.81, .82) |
|  | Dementia/Amnestic disorders due to medical conditions | 294 |
|  | Major depressive, Bipolar disorder | 296 (296.00 to 296.89) |
|  | Anxiety disorders as well as:  Conversion disorders (300.11)  Dissociative (300.12 through 300.15)  Factitious (300.15, 300.19)  Dysthymic disorder (300.4)  Depersonalization, body dismorphic, hypochondriasis, somatoform (300.6, 300.7, 300.81)  Unspecified mental disorder (300.9) | 300 |
|  | Cyclothymic disorder | 301.13 |
|  | Personality disorders | 301  Except 301.13 |
|  | Sexual dysfunction, pedophilia, paraphilia, etc. | 302 |
|  | Vaginismus (not due to a general medical condition) | 306 |
|  | Eating disorders, tic disorder, Tourette’s, insomnia, sleep disorders | 307 |
|  | Acute stress disorder | 308.3 |
|  | Adjustment disorders as well as PTSD (309.81) | 309 |
|  | Personality change due to medical condition | 310 (inclusive) |
|  | Depressive disorder, nos | 311 |
|  |  |  |
|  | Impulse control disorders (e.g., kleptomania, conduct disorder, etc.)* | 312 |
|  | Other disorders usually dxed in infancy, etc. (e.g., selective mutism, oppositional defiant disorder, etc.)* | 313 |
|  | ADHD* | 314 |
|  | Psychological factor affecting a medical condition | 316 |
|  | Mental retardation (317-319) | EXCLUDED |
|  | All codes after 319 | EXCLUDED |
|  | **OMHRS provision diagnosis (use only if no DSM-IV diagnosis)** | |
|  | DSM: various | If 1 in (Q1B, Q1C, Q1F to Q1P) |
| **Substance-related, addictive disorders** | **OHIP** | |
|  | Alcoholic psychosis, DTs, Korsakov’s | 291 |
|  | Drug psychosis | 292 |
|  | Alcoholism; alcohol intoxication/dependence | 303 |
|  | Drug dependence, drug addiction | 304 |
|  | Drug, tobacco abuse | 305 |
|  | **ICD-9** | |
|  | Alcohol, drug-induced mental disorders | 291, 292 |
|  | Alcohol, drug dependence | 303, 304 |
|  | Non-dependent drug abuse | 305 |
|  | **ICD-10** | |
|  | Mental disorders due to psychoactive substance use | F1 |
|  | **DSM-IV** | |
|  | Alcohol-related/induced conditions | 291 |
|  | Other substance-related withdrawal (amphetamines, opiods, sedatives, etc.) | 292 |
|  | Alcohol intoxication, dependence | 303 (inclusive) |
|  | Other substance dependence,abuse | 304  305 |
|  | **OMHRS provision diagnosis (use only if no DSM-IV diagnosis)** | |
|  | DSM: Substance-related disorder | Q1D = 1 |
